# Strain-specific copy number variation in the intelectin locus on the 129 mouse chromosome 1

**DOI:** 10.1186/1471-2164-12-110

**Published:** 2011-02-16

**Authors:** Zen H Lu, Alex di Domenico, Steven H Wright, Pamela A Knight, C Bruce A Whitelaw, Alan D Pemberton

**Affiliations:** 1 The Roslin Institute and Royal (Dick) School of Veterinary Sciences, University of Edinburgh, Roslin, Midlothian, UK

## Abstract

**Background:**

C57BL/6J mice possess a single *intelectin (Itln) *gene on chromosome 1. The function of intelectins is not well understood, but roles have been postulated in insulin sensitivity, bacterial recognition, intestinal lactoferrin uptake and response to parasites and allergens. In contrast to C57BL/6J mice, there is evidence for expansion of the *Itln *locus in other strains and at least one additional mouse *Itln *gene product has been described. The aim of this study was to sequence and characterise the *Itln *locus in the 129S7 strain, to determine the nature of the chromosomal expansion and to inform possible future gene deletion strategies.

**Results:**

Six 129S7 BAC clones were sequenced and assembled to generate 600 kbp of chromosomal sequence, including the entire *Itln *locus of approximately 500 kbp. The locus contained six distinct *Itln *genes, two *CD244 *genes and several *Itln*- and *CD244*-related pseudogenes. It was approximately 433 kbp larger than the corresponding C57BL/6J locus. The expansion of the *Itln *locus appears to have occurred through multiple duplications of a segment consisting of a full-length *Itln *gene, a *CD244 *(pseudo)gene and an *Itln *pseudogene fragment. Strong evidence for tissue-specific distribution of *Itln *variants was found, indicating that *Itln *duplication contributes more than a simple gene dosage effect.

**Conclusions:**

We have characterised the *Itln *locus in 129S7 mice to reveal six *Itln *genes with distinct sequence and expression characteristics. Since C57BL/6J mice possess only a single *Itln *gene, this is likely to contribute to functional differences between C57BL/6J and other mouse strains.

## Background

Intelectins are glycoproteins with an approximate subunit size of 37 kDa, that have been described in mammals, fish and amphibians. The genome sequence of the sea squirt *Ciona intestinalis *[1] shows the presence of closely-related genes, indicating that intelectins arose early in chordate evolution. In mammals, intelectins have so far been described in humans [2-4], mice [5,6], sheep [7], cattle [8] and pigs [9], although interestingly, the dog genome [10] apparently lacks any intelectin genes.

The three-dimensional structures of intelectins have not yet been elucidated. Much of the predicted protein sequence is highly conserved across species, including eight cysteine residues and a fibrinogen-like domain [11]. There has been significant interest in the functions of intelectins, which appear to be many and varied. A Ca^2+^-dependent galactose-specific lectin activity was first detected in *Xenopus *intelectins, such as XL35 [12], which subsequently led to the naming of the family as "intelectins" (intestinal lectins) following the detection of a closely-related gene in mouse small intestinal Paneth cells by Komiya *et al*. [5]. Due to the cellular localisation of expression of this gene, currently denoted *Itln1 *or *Itlna*, a role in innate immunity was postulated.

Human and mouse intelectins were shown to bind lactoferrin [3,13], leading to the alternative nomenclature of "intestinal lactoferrin receptor" (*Lfr*). Additionally, transcripts identical to human intelectin-1 (Itln1) were detected in omental fat tissue, and termed omentin by Yang *et al*. [14]. These authors and others [15] have investigated the metabolic significance of Itln1, which is present in blood plasma and is thought to function as an adipocytokine. Circulating levels are inversely correlated to body mass index [15] and to occurrence of type-1 diabetes mellitus [16]. A single nucleotide polymorphism in *Itln1 *has also been implicated in susceptibility to Crohn's disease [17], and a closely-linked coding SNP was also found to be associated with incidence of asthma [18].

Intelectin expression in the gut and lung mucosa is known to be highly up-regulated in the immune response to parasitic infections and Th2-cytokine dominated allergic responses [19-22]. Whilst *Itln1 *is expressed in Paneth cells at a high level in normal mouse small intestine, infection of BALB/c mice with the small intestinal dwelling helminth *Trichinella spiralis*, induced *de novo *expression of an additional gene termed intelectin-2 (*Itln2*) in goblet cells, which was secreted into the mucus layer [6]. This gene, alternatively termed *Itlnb*, is absent from the reference C57BL/6J genome, and it was suggested that lack of *Itln2/Itlnb *may be partially responsible for the delayed expulsion of *T. spiralis *in C57BL/6J mice compared to BALB/c [6].

In a subsequent genome-wide screen for gene copy number variation (CNV), Graubert *et al*. [23] highlighted the *Itln *locus on distal chromosome 1 as a hotspot of gene duplication in most mouse strains analysed. Gene duplication [24] represents an evolutionary mechanism whereby populations can adapt to increase the effective dosage of an advantageous gene in the face of a particular selection pressure, such as the burden of intestinal parasites. Once fixed, duplicated genes (paralogs) can evolve distinct functions. In the context of responses to parasites, a notable example of gene duplication in the mouse is at the mast cell proteinase locus on chromosome 14 [25]. Mast cell proteinases contribute anti-parasite effector mechanisms, and the gene *Mcpt1 *has been shown to enhance expulsion of *T. spiralis *[26].

In this study, we aimed to sequence the *Itln *locus in a non-C57BL/6J strain, in order to characterise the nature and extent of duplication of mouse *Itln *genes. We focussed on the 129S7 mouse, as a BAC library was available for this strain [27], which is commonly used in gene deletion studies, and this information would enable the design of future specific *Itln *knockout strategies.

## Results

### Sequencing of 129S7 *Itln *locus

**Figure 1:**
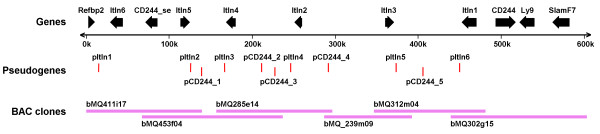
**Schematic representation of the 129S7 *Itln *locus**. Genomic DNA from six overlapping 129S7 BAC clones was sequenced and assembled as described in Materials and Methods to give a ~600 kbp final assembly. The locations of genes and pseudogenes are indicated.

The *Itln *locus on chromosome 1 of the Celera mouse assembly [28] shows multiple copies of *Itln *genes as compared to that of the C57BL/6J reference genome. However, it is an assembly consisting of a mix of 5 different mouse strains (129X1/SvJ, 129S1/SvImJ, DBA/2J, A/J and C57BL/6J) and there are about 43 kbp of gaps on this locus. To improve on the annotation and resolve the exact structure of the 129S7 *Itln *copy number variations, 4 flanking bacterial artificial chromosome (BAC) clones were sequenced to a depth of ~4.7× using the conventional Sanger sequencing method while the remaining middle two were sequenced to a much greater depth of ~300× using the Illumina paired-end sequencing method. In view of the expected repetitive nature of the *Itln *locus, the use of this tiling path sequencing strategy was necessary to minimise errors in the assembly. Nevertheless, several gaps estimated (based on the size of the individual BAC clones) to be less than 10 kbp in total are yet to be bridged. Gap closure over repeat elements with high level of sequence identity remains computationally challenging. The final assembly of the 6 BAC clones yielded a scaffold of about 603 kbp (Figure 1); adding approximately 16 kbp of sequences to the corresponding Celera assembly. The overall sequence identity between the two is about 97%. However, there exist variable regions with sequence identity as low as 60% (Additional file 1) suggesting potential errors in the assembly using mixed strains of mouse. The sequence and annotations of the scaffold have been deposited in the GenBank with the accession number HM370554 and a graphical representation is also available at http://www.ncbi.nlm.nih.gov/nuccore/300085578?report=graph.

### Analysis of the 129S7 *Itln *locus

**Figure 2:**
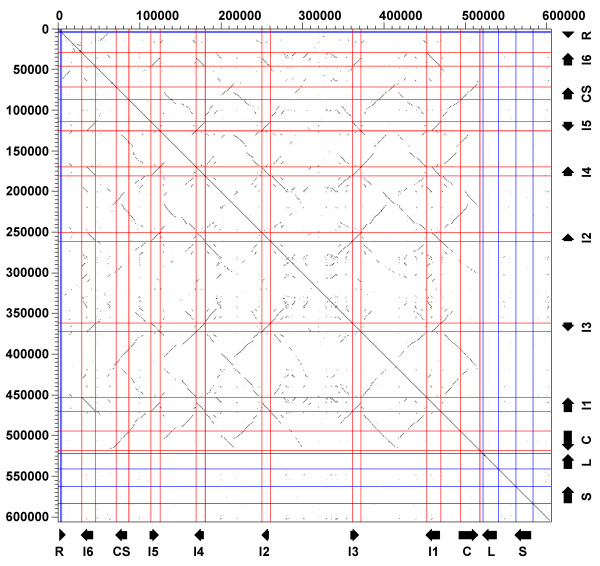
**Tandem duplication and inversion of *Itln *and *CD244 *genes**. Dotplot analysis of 129S7 locus against itself. Duplicated regions are plotted with lines parallel (tandem duplications) and perpendicular (inverted duplications) to the straight diagonal line. The locations of genes *Refbp2 *(R), *Itln6 *(I6), *CD244_SE *(CS), *Itln5 *(I5), *Itln4 *(I4), *Itln2 *(I2), *Itln3 *(I3), *Itln1 *(I1), *CD244 *(C), *Ly9 *(L) and *SlamF7 *(S) are indicated.

A dotplot analysis (Figure 2) was first used to pinpoint regions of high similarity where the *Itln *genes may be located. Six complete or partial ~62 kbp tandemly or invertedly duplicated segments could be identified on the locus. Each of these segments is found to be flanked by murine endogenous retroviral (ERV) elements which have been known to mediate the host recombination [29]. These ERV elements may well serve as the breakpoint for the recombination and expansion of the *Itln *locus. It is perhaps no coincidence that about 43% of the 129S7 *Itln *locus is made up of repeat elements. This is significantly denser than the average 37.5% observed for the whole genome in the C57BL/6J mouse [30].

In addition to the flanking genes (*Refbp2*, *Ly9 *and *Slamf7*), the ~500 kbp *Itln *locus has been predicted to be made up of 5 full-length *Itln *variants (*Itln1-2,4-6*), one truncated *Itln *(*Itln3*), 6 pseudo-*Itln*, one *CD244 *variant *CD244_SE*, one full-length *CD244 *and 5 pseudo-*CD244 *(Figure 1 & Additional file 2). The 6 *Itln *genes can be further classified into two categories based on their sizes of either ~11 or ~17 kbp; with *Itln1 *and *Itln6 *having additional LINE/L1 retrotransposable elements inserted into their respective intron 7. In fact, variations also exist in the organisation of the pseduogenes on each of the duplicated segments; with some having different exons removed.

**Figure 3:**
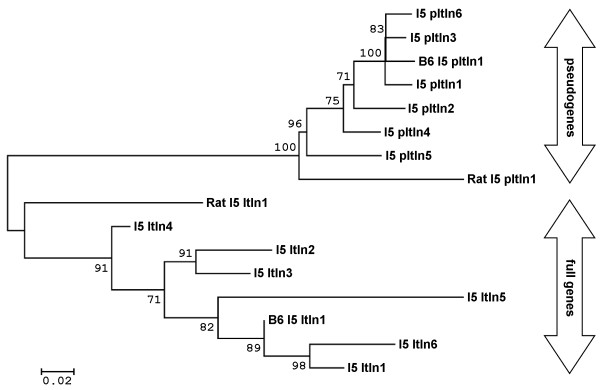
**Phylogenetic analysis of the *Itln *genes and pseudogenes in 129S7, C57BL/6J and rat**. The unrooted phylogenetic tree was constructed based on the sequences of Intron 5 (I5) which range from about 400 bp to 3.6 kbp. Numbers at the beginning of nodes represent the percentage bootstrap support. The full-length (Itln)- and pseudo-genes (pItln) separate into two distinct clades. All C57BL/6J-derived (pseudo)genes are prefixed "B6".

To further investigate the evolutionary basis of the seemingly active segmental duplication, an unrooted neighbour-joining tree (Figure 3) was constructed using intron 5 and its equivalent in all the *Itln *genes and pseudogenes, respectively. With less selection pressure than the exons, introns are known to mutate more rapidly and uniformly; making them an informative phylogenomic marker. The tree shows that the full-length genes and pseudogenes are clearly separated into two distinct clades. The clustering of the pseudogenes suggests that they probably propagate by serial duplication of an existing pseudogene which may already be present within the duplicated segments together with its full-length gene. Detailed sequence analysis of each of the introns shows that the insertions of ERV and/or LINE repeats probably account for the branching of each genes and pseudogenes.

Although all 8 exons of the *Itln *genes can be mapped onto the 6 variants and their predicted mRNA sequences are highly conserved (94 to 97% sequence identity), they are predicted to be expressed and probably regulated differently. Both *Itln3 *and *Itln4 *transcripts are found to contain an early stop codon. While this is most likely to result in a truncated Itln3, the presence of potential ribosomal -1 frameshift sites identified further upstream of the stop codon at positions 175 and 177 is predicted to allow the translation of the full-length Itln4 protein to proceed. Sequence conservation among the 5 predicted full-length Itln proteins ranges from 91 to 96%. Not surprisingly, similar conservation is also observed when comparing the variants with the Itln1 [GenBank:NP_034714] of C57BL/6J; with the Itln1 from both strains being identical to each other on the protein level. However, higher degrees of variation are found among the 5 Itln variants and the predicted Celera homologues; with the protein sequence identity ranges from 71 to 100% (Additional file 1). Two of Celera's *Itln *variants may also contain sites for ribosomal -1 frameshifting. The *Itln1 *and *Itln2 *genes of 129S7 are found to be identical to Celera's *Itlna *[GenBank:NP_034714] and *Itlnb *[GenBank:NP_001007553] respectively. The observed variation in the remaining *Itln *variants is probably the result of a mixed *Itln *population derived from the 5 mouse strains.

*CD244_SE *was initially predicted to be a *CD244 *pseudogene due the lack of the terminal exon 9. However, a closer inspection has resulted in the identification of a potential internal splicing site in the predicted exon 3. Comparison of all the probable coding sequences derived from this internal splicing site has led to the prediction of a protein which shares 65% identity with the soluble form of rat CD244 [31]. This novel protein is made up of 4 exons which are highly similar (~90% conservation) to that of CD244; with part of exon 3 spliced to exon 5. On the other hand, it is also interesting to note that the CD244 protein sequence of 129S7 and Celera [GenBank:XP_001003781] are almost identical with 99% sequence identity but they share only 89% sequence identity with that of C57BL/6J [GenBank:NP_061199].

All the pseudogenes contain exons that share at least 70% sequence identity with their full-length counterparts. None of them contains the full set of exons and multiple stop codons are often spread across them.

### Southern blot analysis

**Figure 4:**
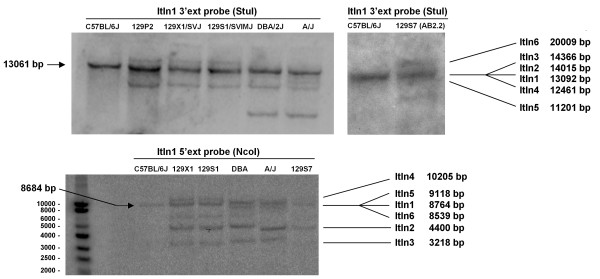
**Southern blot detection of *Itln *locus in C57BL/6J and 129-related mouse strains**. A combination of 5' and 3' external probes enables the variants of different sizes to be detected and they were in agreement with that predicted (as indicated) computationally. *Stu *I digestion also resulted in an unspecific ~8 kbp fragment which contained sequence that shared some homology with the 3' probes. Polymorphisms in other 129-related strains could potentially result in unspecific hybridisation of the probes.

Southern blot analysis (Figure 4) was conducted to experimentally verify both the assembly and the dotplot analysis on the number of duplicated segments. Using probes (Additional file 3) that hybridised to the 5' and 3' end of the *Itln *genes, fragments gave a banding pattern that was consistent with that predicted from the *in silico *restriction analysis of the locus. In addition to the 129S7 strain, Southern blotting was also carried out on C57BL/6J, 129X1, 129S1, DBA2/J and A/J mice (Figure 4). All the 129 substrains also showed a banding pattern that was consistent with restriction fragments predicted from the sequenced 129S7 *Itln *locus. DBA2/J and A/J strains showed a similar pattern, but with the appearance of a prominent extra band with the 3' probe. Although it is highly likely that these four mouse strains share a similar genomic organisation at the Itln locus with the 129S7 substrains, differences observed in the Southern analysis should further warrant a note of care when using the mixed assembly of Celera's mouse genome. The C57BL/6J strain consistently gave a single band, as expected.

### *Itln *CNVs in mouse

**Figure 5:**
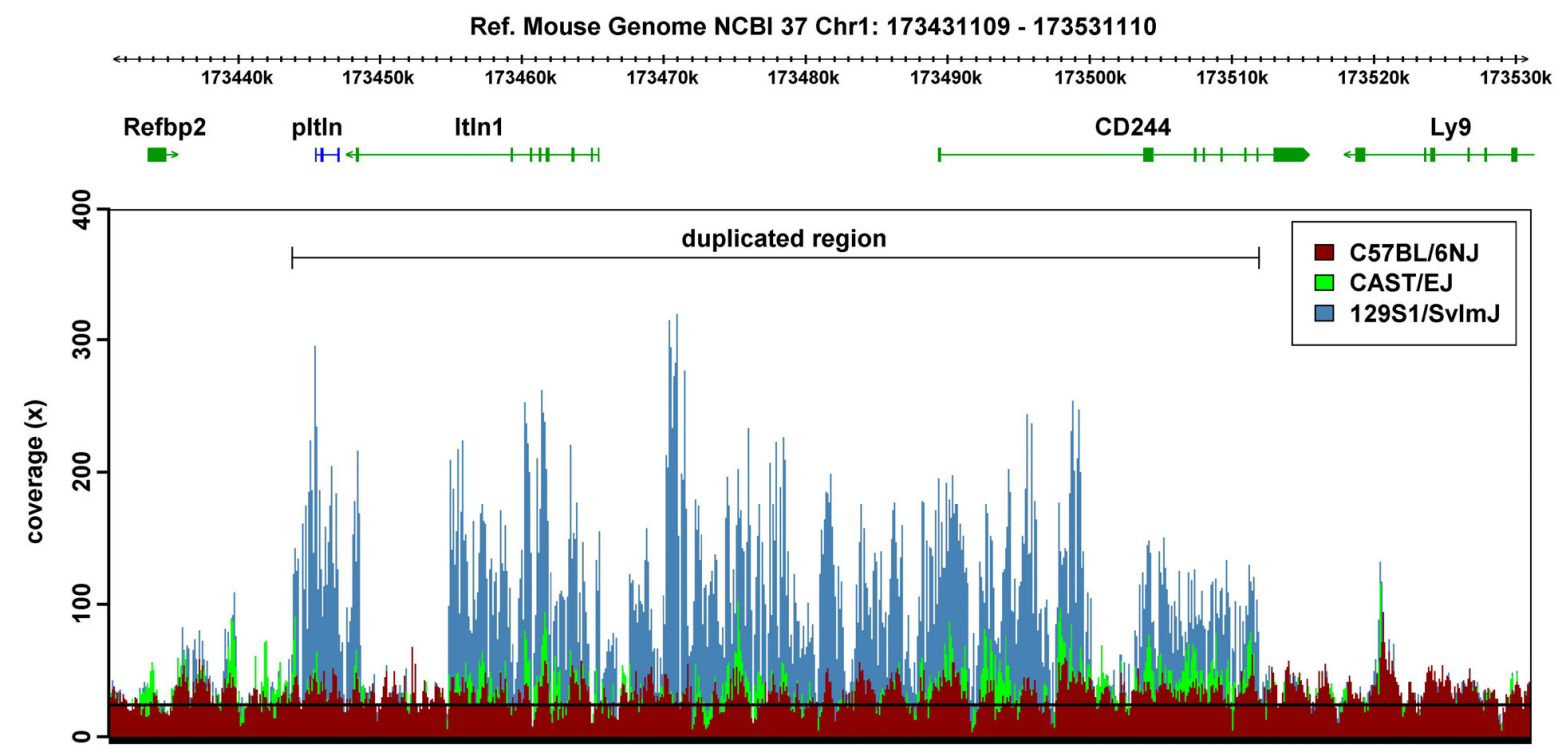
**Evidence for copy number variation in three mouse strains**. Coverages of the paired-end reads for the three strains, C57BL/6NJ (red), CAST/EiJ (green) and 129S1/SvImJ (blue), were plotted along the *Itln *locus of the reference mouse genome and they were superimposed on top of one another. The mean mapped coverage (horizontal line) of the three genomes is about 20x. Coverage of C57BL/6NJ's *Itln *locus was consistent with the mean mapped genome coverage while those of the other 2 strains were expanded to different degrees. Reads that were mapped onto repeat elements were reported once and resulted in regions showing coverages similar to the mean mapped genome coverage.

To date, 5 genome-wide CNV studies of varying degrees of resolution have been carried out in different mouse strains [23,32-35]. However, none has specified the exact copy number of the *Itln *genes. Recently, the Wellcome Trust Sanger Institute has sequenced 17 different mouse strains using the Illumina next generation sequencing technology. With the knowledge that there are 6 *Itln *genes in the 129S7 mouse and that it has a similar Southern pattern to the other 129 substrains, an attempt to identify the *Itln *CNVs in these strains was done by comparing the sequencing coverage across the *Itln *locus (Figure 5 & Additional file 4). During the mapping of these reads against the C57BL/6J reference genome, reads are expected to collapse together to increase the mapping coverage at segments where copy number gains are located. This gain can be quantified by the log_2 _ratio of the coverage at a particular position; with for example a single (heterozygous) copy gain represented as log_2_(3/2) = 0.58. A locus duplicated once in a homozygous inbred strain would be represented as log_2_(4/2) = 1. Such a comparison is in fact analogous to CNV detection using the array comparative genomic hybridisation (aCGH) method [36].

Relative to the single *Itln *locus in the C57BL/6J reference mouse, an approximately 62 kbp segment containing the *Itln *gene has apparently been duplicated several times in all the 16 strains investigated. Although the log_2 _ratio of the CAST/EiJ strain falls close to the threshold (Additional file 4, Figure S3a), the consistently higher than reference coverage across the *Itln *locus (Figure 5) suggests that the segment may actually be duplicated once. The log_2_ratio for the 129S1/SvImJ strain vs C57BL/6J varies between 1.8 and 2.7 over the *Itln *locus (Additional file 4, Figure S3a), well above the threshold for calling gene amplication, and suggesting 4 to 6-fold gain of the locus compared to C57BL/6J. Similar gain is also observed in the other strains (result not shown).

### Evidence for expression of mouse *Itln *variants

Polymerase chain reaction analysis using common primers which amplified the *Itln *cDNA from all mouse tissues sampled, gave a product of the expected size (301 bp). Restriction enzyme analysis (Additional file 5) revealed variation in patterns across the different tissue samples, indicating heterogeneity of *Itln *variant expression. PCR products were sequenced to confirm the identities of variants expressed in different tissues (summarised in Table 1). Briefly, *Itln2 *was expressed in trachea and *Itln1 *was the dominant form expressed in small intestinal tissues, whereas *Itln6 *was dominant in stomach and caecum. Since there was evidence for expression of both *Itln2 *and *Itln6 *in colon, the PCR product was cloned and 48 clone sequences were obtained. Twenty six clones were consistent (>98% identity) with *Itln2 *and 12 were consistent with *Itln6*. The remaining 10 sequences contained ambiguities and/or were not consistent with any of the six known *Itln *variants.

**Table 1:** *Itln *variants identified in various mouse tissues.

Tissue	*Itln *variant identified
Trachea	*Itln2*

Stomach	*Itln6*

Duodenum	*Itln1*

Jejunum	*Itln1*

Ileum	*Itln1*

Caecum	*Itln6*

Colon	*Itln2 *&*Itln6*

Summary of the RT-PCR and sequencing of pan-*Itln *PCR products show tissue specific expression of the variants. Similar results were obtained with 129S2 and 129P2 tissues (Additional file 5). These results do not preclude the presence of additional *Itln *variants in any tissue.

### Detection of putative Nkx3.1 transcription factor binding sites in *Itln *promoter regions

**Figure 6:**
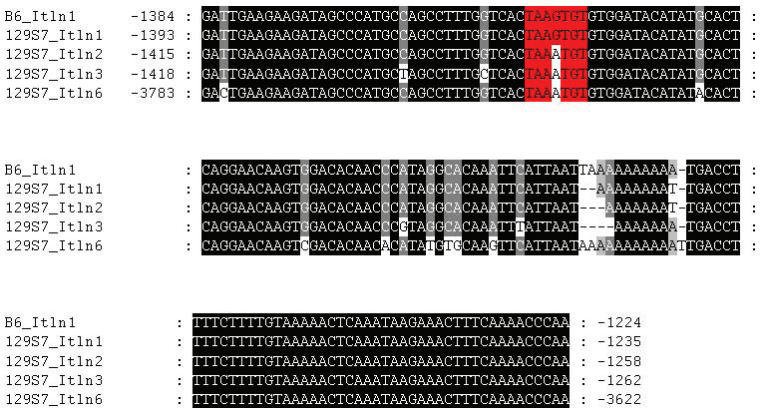
**Comparison of promoter regions of *Itln *genes**. The Nkx3.1-binding promoter region 5 sequence, identified by Mogal *et al*. [37], was detected in the promoters of *Itln1 *in C57BL/6J (*Itlna*) and four *Itln *genes in 129S7 (*Itln1, Itln2, Itln3 *and *Itln6*). The canonical Nkx3.1 binding sites on the promoters are highlighted.

In C57BL/6J, the so-called "Int5" promoter region of *Itln1*, which we have renamed Promoter Region 5 (Pr5) to avoid confusion, has been found to harbour the binding site for the transcription factor Nkx3.1 [37] at position -1348 to -1343. The cognate DNA binding sequence for Nkx3.1 was determined by Steadman *et al*. [38] to be TAAGT(A/G). This Pr5 promoter region is found to be conserved in *Itln1*, *Itln2*, *Itln3 *and *Itln6 *of the 129S7 mouse (Figure 6). Since Itln3 is predicted to be a truncated protein, it was excluded in the subsequent search for the Nkx3.1 binding site. Putative binding sites on the promoter of *Itln1*, *Itln2 *and *Itln6 *are predicted to be located at positions -1357 to -1351, -1379 to -1373, and -3747 to -3741 respectively (Table 2).

**Table 2:** *In silico *prediction of Nkx3.1 binding sites in 129S7 *Itln *promoters.

*Itln*	Model	Score	Relative score	Start	End	site sequence
*Itln1*	NKX3-1	8.333	0.883282576	-1357	-1351	TAAGTGT

*Itln2*	NKX3-1	5.085	0.766546505	-1379	-1373	TAAATGT

*Itln6*	NKX3-1	5.085	0.766546505	-3747	-3741	TAAATGT

JASPAR scan for the conserved Nkx3.1 motif in the 5 kbp upstream (represented by the negative positions) promoter regions.

However, the "TAAGTG" motif is only conserved between the *Itln1 *promoter of C57BL/6J and 129S7. A single bp variation results in a "TAAATG" binding motif on the promoters of *Itln2 *and *Itln6 *which may in turn affect the binding affinity for Nkx3.1.

## Discussion

We present here the sequence of the *Itln *locus on mouse chromosome 1 in a non-C57BL/6J mouse strain (129S7). Multiple duplications of *Itln *and *CD244 *genes/pseudogenes have resulted in the expansion of the locus in comparison to that found in the reference C57BL/6J mouse. Nearly half of this expanded *Itln *locus comprises repeat sequence elements. It is likely that the presence of flanking ERV and LINE elements was involved in the duplications and inversion of the *Itln *locus; and that the events may be relatively recent and active [29,39].

The present data allows us to unequivocally define the genetic architecture of the *Itln *locus in the 129S7 genome. It bears considerable similarity to that obtained in the Celera mouse genome but with reduced homology over the coding regions. This is most likely due to the Celera sequence being a composite of 5 different strains and the difficulty in resolving repetitive sequences; and hence not necessarily representative of any one strain. It has been estimated that as much as 57% of highly identical segmental duplications in the mouse genome were potentially misassembled and that segmental duplications/CNVs make up 1.7-2.0% of the mouse genome [40]. We have overcome this pitfall by a combined strategy of tiling path sequencing of BAC clones and Southern verification.

Furthermore, our Southern blot data suggested that other non-C57BL/6J strains also have a similar structure. In support of this, not only have a number of previous genome-wide studies on the structural variations of the mouse genome identified this locus as a hotspot of recombination and CNV, the ENSEMBL annotated Itln protein family ENSFM00250000003313 also reports variations in the copy number of the *Itln *gene in different animal species. The majority of the 17 mouse strains show evidence of expansion at this locus too, raising the question as to whether the *Itln *locus has been deleted in the C57BL/6J and related strains, or alternatively expanded in a common ancestor to the 129S7 and other non-C57BL/6J strains.

It was not known whether CNV at the mouse *Itln *locus confers a simple dosage effect, or alternatively, whether each variant plays a different functional role. We found that in contrast to the C57BL/6J mouse strain, which expresses a single *Itln1 *gene [30], 129S2 and 129P2 mice exhibited site-specific expression of at least three different *Itln *variants along the gastrointestinal tract. Such differential tissue expression of *Itln *variants has also been observed in the channel catfish [41]. This highly selective expression pattern and the minor amino acid sequence differences between the *Itln *variants suggest that these duplicated genes may be of functional significance at the different sites of expression. It has been suggested that intelectins play a role in modifying mucus properties, and therefore site-selective expression of variants may be important in the same way that mucosal trefoil factor variant expression tends to be co-ordinated with specific mucin types in different regions of the gastrointestinal tract [42]. Similarly, sheep intelectin variants have been found to exhibit site-specific expression differences between airways, gastric stomach (abomasum) and small intestine [7]. However, the consequences to C57BL/6J mice, if any, of lack of expression of Itln2 and Itln6 in the GI tract, remain to be established.

It has been shown that a functional Nkx3.1 transcription factor binding site is located in the mouse *Itln1 *promoter [37]. Nkx3.1 is highly expressed in the prostate, where it is believed to function as a tumour repressor gene [43], and regulates Itln1 expression in prostate epithelial cells [44]. Haploinsufficiency in *Nkx3.1*, where only a single functional allele of the gene is present, was found to result in reduced expression of a range of dosage-sensitive genes in the mouse prostate, including *Itln1*, expression of which was essentially lost in *Nkx3.1*^+/- ^mice [44]. Importantly, Itln1 expression was itself shown to suppress prostate cell growth [37], and thus Itln1 appears to be an effector of prostate cancer repression. It was of interest therefore to determine the occurrence of Nkx3.1 binding sites in all six *Itln *genes described here. The Pr5 region which was found to contain the Nkx3.1 binding sequence TAAGTG [38] was present in the promoter regions of four of the six *Itln *genes but the binding region was mutated to TAAATG in all but *Itln1*, suggesting that although *Itln1 *probably remains under the transcriptional control of Nkx3.1 in 129S7 mice, the same is not necessarily true for the other *Itln *variants.

Importantly, an adipocytokine [14] role has been ascribed to human Itln1 (omentin-1), which can be detected in blood plasma and serum. Estimates for normal human serum Itln1 concentration vary from 20 ng/ml [45] to over 100 ng/ml [14]. Recombinant Itln1 has been shown to stimulate insulin-dependent glucose uptake by adipocytes *in vitro *[14], and its expression in visceral fat and in the circulation is negatively correlated with body mass index and waist circumference [46]. Weight loss and aerobic exercise were associated with significant increases in serum Itln1 levels [47] and a corresponding reduction in cardiometabolic risk factors.

With reference to the comparative metabolic significance of *Itln *expression in mice, Orozco *et al*. [48] investigated the influence of CNV on metabolic traits using a combined genomic - metabolomic approach in the C57BL/6J (B6) and C3H/HeJ (C3H) strains, and in B6xC3 H crosses. Hotspots of copy number variation in chromosomes 1, 4 and 17 were associated with metabolic traits, and specifically, CNV in *Itln1 *(*Itlna*) was linked with weight, triglycerides, adiposity, glucose and insulin level [48]. *Itln *mRNA expression level, as detected by Agilent microarray analysis [48], was significantly elevated in the high copy number genotype (C3H), when assayed in adipose, brain and liver tissue. The detailed characterisation of the *Itln *locus described here provides new specific candidates for further investigation of the role of *Itln *genes in mouse models of metabolic disorders.

In addition to *Itln*, the *CD244 *gene sequence is also amplified within the 129S7 locus. Specifically, this study has identified a putative *CD244 *variant, analogous to the secreted CD244 variant previously described in the rat [31]. However, we found no evidence of expression of the corresponding transcript by RT-PCR in mouse small intestine (data not shown), so it is not clear whether it is actively transcribed in this mouse strain. *CD244 *(also known as *2B4*) is a member of the SLAM family of cell surface receptors, which are present as a gene cluster adjacent to the *Itln *locus on mouse and human chromosome 1. A range of lymphocytes, including natural killer (NK) cells and CD8^+ ^T-cell subsets have been shown to express CD244, which is the high affinity counter-receptor for CD48 (for a review, see [49]). While engagement of human NK cell CD244 with CD48 results in enhanced killing of CD48-expressing cells [50], the situation appears more complex in mice, where both activating and inhibitory roles have been demonstrated [51]. It is known that C57BL/6J mice possess a single *CD244 *gene, expressed as long and short splice variants, which are distinct from CD244 cDNAs amplified from other mouse strains [52]. The potential expression of a soluble CD244 variant suggested by this study, may introduce an additional level of regulation of lymphocyte interactions in non- C57BL/6J -related strains, and may therefore contribute to immunological differences between C57BL/6J and other strains.

## Conclusions

To conclude, we have determined the sequence for essentially the complete *Itln *locus in the 129S7 mouse, as a prototype for non-C57BL/6J mouse strains. This has elucidated the nature of the copy number variation occurring at this locus, arising from tandem duplication of *Itln *and *CD244 *genes. Individual *Itln *genes showed strong tissue expression specificity while most duplicated *CD244 *genes were non-functional.

## Methods

### Identification and sequencing of *Itln*-containing BAC clones

The *Itln *locus of the 129/Sv mouse was characterised by sequencing BAC clones derived from the AB2.2 ES cell of the 129S7/SvEvBrd-Hprt^b-m2 ^substrain [27]. Since the reference C57BL/6J mouse genome contains only a single copy of the *Itln *gene (Additional file 2), two BAC clones flanking the gene were first identified from the BAC end sequences aligned to the ENSEMBL Mouse Assembly (ENSEMBL release 46, NCBI m36) [53]. A tiling path of candidate BAC clones moving towards each other from the two ends of the locus (Figure 1) was then built by selecting BAC clones with end sequences that matched to the sequenced ones. Two iterations of blast search were done to identify these matching clones; an initial identity cut-off of 90% was applied, followed by quality trimming of the filtered sequences and a final round of blast with 98% identity cut-off. PCRs to check the presence of *Itln *were also performed to further ensure the correct candidates were picked. A total of six overlapping BAC clones (bMQ411i17, bMQ453f04, bMQ285e14, bMQ_239m09, bMQ312m04 and bMQ302g15) spanning the entire *Itln *locus were purchased from Geneservice (UK). BAC clones were grown in LB broth containing chloramphenicol (12.5 μg/ml) and DNA was purified using a proprietary kit (NucleoBond^® ^BAC 100, Macherey-Nagel). Four shortgun libraries with insert size of about 2 kbp were constructed from the four respective flanking clones (bMQ411i17, bMQ453f04, bMQ312m04 and bMQ302g15) and sequenced by GATC Biotech (Konstanz, Germany) using the ABI Big Dye Terminator Mix v3.0 in a ABI 3730 sequencing machine. Gap closures for these four clones were achieved by primer walking. 36 bp paired-end next-generation sequencing using the Illumina Genome Analyzer II was carried out by ARK-Genomics (Roslin, UK) on the two remaining middle clones (bMQ_239m09 and bMQ312m04).

### Sequence assembly

Sequences from each of the four shortgun libraries were assembled using Phred (v 0.020425.c)/Phrap (v1.080812)/Consed (v15.0) [54] into their respective scaffolds. Parameters for the assembly were set to high stringency: vector scanning, 32bp trimming of all 5' ends, minimum Phred score of 20, minimum length of matching word increased to 30, and level of contigs merging stringency set to the highest. Contaminating contigs from *E. coli *were removed by blasting them against the NCBI's *E. coli *genomes [55]. All assembled contigs were also manually inspected to correct for errors due to duplicated segments and repeats; with special attention paid to regions having higher than 97% cross_match (v 1.080812) [54] identity. They were ordered into a single scaffold for each of the libraries with the aid of the forward/reverse pairing data and locations of the predicted coding sequences.

A strategy involving three iterations of mapping against reference sequences, *de novo *sequence assembly and a final scaffold construction was employed to assemble the remaining two BAC clones. The 4.8 million 36 bp Illumina paired-end reads were adaptor- and quality-trimmed to 32 bp with the fastx toolkit (v0.0.11) [56] to ensure only reads with phred scores higher than 15 were kept. These processed reads were first mapped against the contaminating *E. coli str. K-12 substr. DH10B *[GenBank:NC_010473] reference genome and pBACe3.6 [GenBank:U80929] cloning vector using MAQ (v0.7.1) [57]. The 2.4 million clean unmapped reads next underwent a second round of mapping, again with MAQ, to remove sequences overlapping with the known flanking upstream BAC clones. Reads which were unmapped, containing indels or more than one mismatch and mapping quality score less than 20 amounted to about 1.2 million. They were finally MAQ mapped, allowing 2 mismatches, against the corresponding *Itln *locus of Celera's mouse assembly. Again the unmapped reads were extracted and input into the *de nov*o assembly program, Velvet (v0.7.54) [58] to obtain a set of contigs that should cover the gaps in the MAQ mapped consensus sequence. In addition, *de novo *assembly using Velvet on the 2.4 million reads extracted from the first mapping exercise was also done to facilitate the resolution of errors in the MAQ consensus sequence. The Velvet optimiser script was used and no scaffolding was done. Contigs with exceptionally high or low k-mer coverage values were also discarded. The scaffold for these two BAC clones was constructed by merging together the final MAQ consensus sequence and the two sets of Velvet contigs using the SeqMan module of Lasergene (v8.1). Merging was only allowed where end sequences overlapped for at least 15 bp with 99% identity. Where necessary manual correction was always performed. The final single scaffold of the six BAC clones was also assembled with the SeqMan.

### Annotation

Spidey (v1.40) [59] and Blast2seq (v2.2.20) [60] were used initially to search and predict the gene organisation of the 6 known genes, namely *Refbp2*, *Itln1*, *Itln2*, *CD244*, *Ly9 *and *Slamf7*, on the new 129S7 Itln locus. The reference mRNA sequences and the intron/exon boundaries used were obtained from both NCBI Refseq and ENSEMBL gene transcripts (Additional file 2). Splicing junctions were manually corrected to reflect the exact acceptor/donor sites. Pseudogenes were only annotated when their exons share at least 70% identity to the coding sequences. In addition, the exon-exon junctions of the predicted transcripts were predicted with the RNASPL program of the Softberry web server [61] to check for alternative splicing. Potential frame-shifting in the transcripts were checked with the web tool KnotInFrame [62]. Repeat elements on the locus were identified using the program RepeatMasker (v3.2.7) [63].

### Comparative genomics

Dotplot and blastz analyses between the *Itln *locus of the 129S7 strain and itself and that of the Celera mouse assembly were carried out using dotter (v3.1) [64] and zPicture [65] to gain insights into the structural organisation of the locus. Intelectins of other animals were obtained from the ENSEMBL protein family ENSFM00250000003313. ClustalW (v1.83) [66] was used to align the mRNA and the translated protein sequences of the different intelectins while MUSCLE [67] was the program of choice for the multiple sequence alignment of the genomic and the intronic sequences. For the phylogenetic analysis, the alignment of intron 5 sequences of the full length and pseudo-*Itln *genes from 129S7, C57BL/6J and rat was manually adjusted before being analysed with the MEGA (v5.0 beta) software suite [68]. The tree was constructed using the neighbour-joining methodology with the branch distances computed based on the Kimura-2-parameter model. All ambiguous positions were removed for each sequence pair. The final consensus tree was inferred from 1000 bootstrap replicates. The presence of the *Itln *CNV in 17 other mouse strains (129P2/OlaHsd, 129S1/SvImJ, 129S5/SvEvBrd, A/J, AKR/J, BALB/cJ, C3H/HeJ, C57BL/6NJ, CAST/EiJ, CBA/J, DBA/2J, LP/J, NOD/ShiLtJ, NZO/HiLtJ, PWK/PhJ, Spret/EiJ and WSB/EiJ) was detected by using CNV-seq [36] with the threshold of the log_2 _ratio set to 0.6. Furthermore, the relatively high sequencing coverage, averaging 20.1 fold, of the 17 genomes allows the use of a small sliding window size of about 4 kb and a *p*-value of 10^-5 ^to increase the CNV resolution (results not shown). The zoom-in coverage plots of the identified *Itln *CNV were drawn by the plotrix library of R [69]. Sequencing data of these mice were obtained from the Wellcome Trust Sanger Institute ftp://ftp.sanger.ac.uk/pub/mouse_genomes/.

### Nkx3.1 transcription factor binding sites

The potential binding sites for the Nkx3.1 transcription factor on the 5 kbp (upstream of the transcription start site) promoters of the six 129S7 *Itln *variants and that of the C57BL/6J were predicted using three different web tools, namely JASPAR scan of individual promoters applying 75% relative profile score threshold [70]; zPicture pairwise alignment between C57BL/6J and 129S7 promoters followed by rVista [71] search for conserved TAA[G/A]T[A/G][A/C/T] binding sites; and MEME motif discovery [72]. Results from the three predictions were next compared to identify conserved binding sites that fall on the evolutionary conserved regions conserved between C57BL/6J and 129S7. Where no such region exists between C57BL/6J and 129S7, sites with the highest similarity to the C57BL/6J's TAAGTG motif were picked.

### Southern blotting

Two probes (Additional file 3) were designed to sit on the 5' and 3' end of the *Itln *genes. The probe sequences were selected based on the conserved regions of the 6 *Itln *variants. There were blasted against the reference mouse genome to make sure that they do not hybridise to other parts of the genome. Southern blots were done according to the standard protocols of Southern [73]. Briefly, genomic DNA from 7 mouse strains (C57BL/6J, 129P2, 129S1/SvImJ, A/J, DBA/2J, 129X1/SvJ and 129S7) were digested over night at 37°C with either *Nco *I or *Stu *I for hybridising with either the 5' or 3' probe respectively. DNAs were then separated on 0.8% agarose gel overnight at 20 V before being transferred to the nylon membrane for hybridisation with the radioactive probes.

### Evidence for expression of *Itln *variants

Samples of the following tissues were collected from healthy uninfected mice of the 129S2 and 129P2 strains: trachea, stomach, duodenum, jejunum, ileum, caecum and colon. The samples were collected into RNAlater and subsequently RNA was extracted, reverse transcribed and then amplified by PCR using common primers for all six *Itln *variants (ITLN_all_F: TCAGCTAGCAACTCTCAGCTCCT; ITLN_all_R ACACTAGCCACCAGGGTCCA; 35 cycles, annealing temp: 57°C). PCR products were sequenced and results analysed for evidence of a predominant *Itln *variant, or evidence of a mixture. Additionally, PCR products were digested with restriction enzymes *Hha *I, *Mbo *II and *Hae *III (see Additional file 5 for specificities). The PCR products from 129S2 colon and 129P2 trachea were subjected to TOPO cloning. Positive clones from 129S2 colon (48) and 129P2 trachea (4) were individually sequenced.

## Authors' contributions

ZL performed the sequence assembly/analysis and co-wrote the manuscript. SW and AD performed the experimental procedures. AP and BW designed the study and AP co-wrote the manuscript. All authors have read and approved the final version.

## Supplementary Material

Additional file 1 **Comparison of *Itln *locus from 129S7 mice and the Celera mouse assembly**. Alignment and dotplot showing the differences between the sequenced 129S7 mouse and the corresponding Celera assembly. Click here for file

Additional file 2 **Genes in the *Itln *containing contigs of 129S7, C57BL/6J & Celera mouse**. A list of accession numbers for the coding sequences on the three assemblies. Click here for file

Additional file 3 **5' and 3' probes used in the Southern blot**. Sequences of the Southern probes. Click here for file

Additional file 4 **Detection of *Itln *CNV in non-C57/BL mouse strains using next-generation sequencing**. Plots of log_2 _ratio and sequencing coverage at the *Itln *locus of different mouse strains. Click here for file

Additional file 5 **Restriction enzyme analysis of *Itln *transcripts amplified from various tissues**. Tissue specific expression of Itln variants in 129S2 and 129P2 mouse. Click here for file
