# Supplementary figures and images for: Strain-specific copy number variation in the intelectin locus on the 129 mouse chromosome 1
Source: BMC Genomics. 2011 Feb 16;12:110. doi: 10.1186/1471-2164-12-110 (PMC3048546; doi:10.1186/1471-2164-12-110)

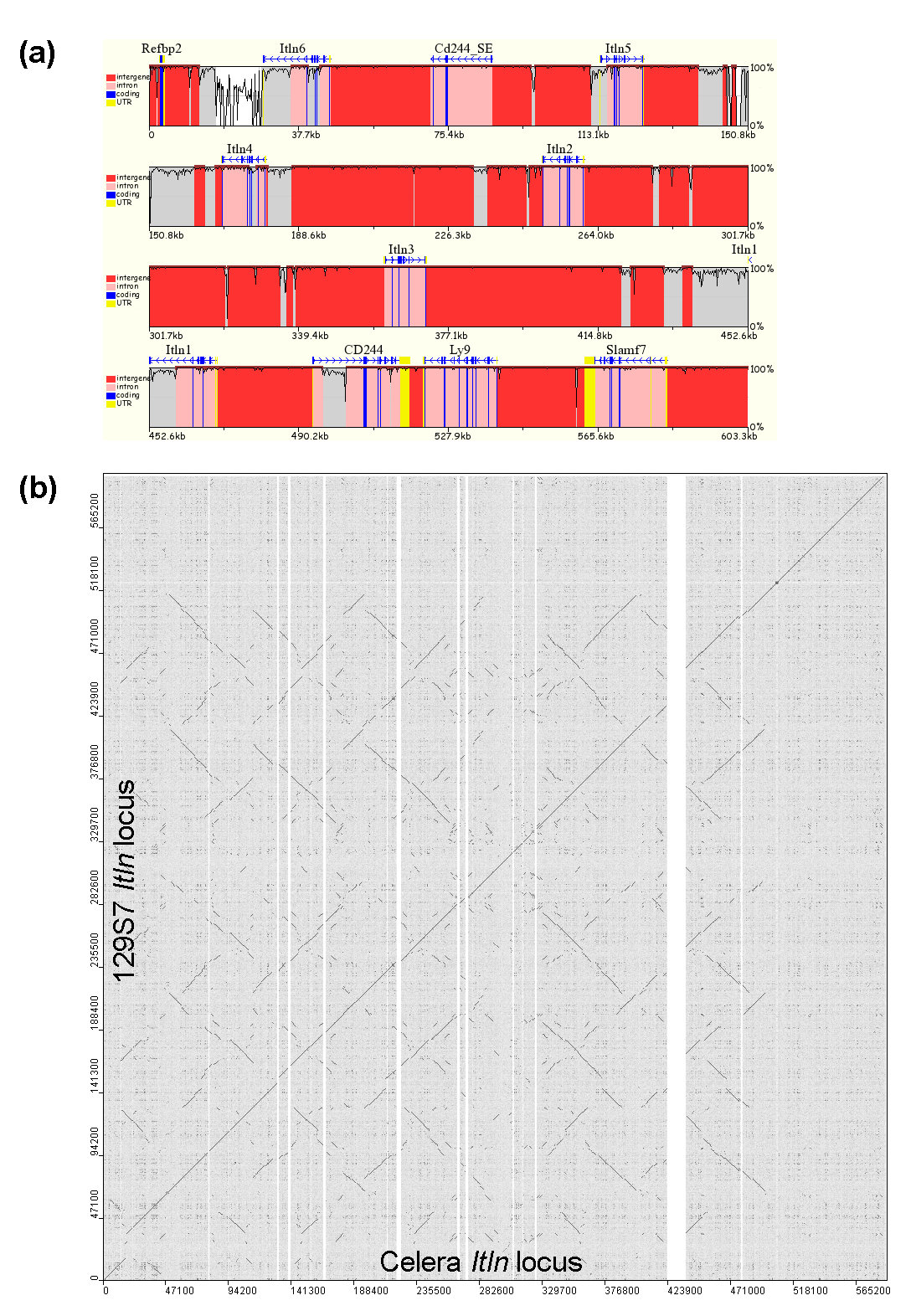

Supplement: Additional file 1 — Comparison of Itln locus from 129S7 mice and the Celera mouse assembly. Alignment and dotplot showing the differences between the sequenced 129S7 mouse and the corresponding Celera assembly. [file 1471-2164-12-110-S1.PNG]

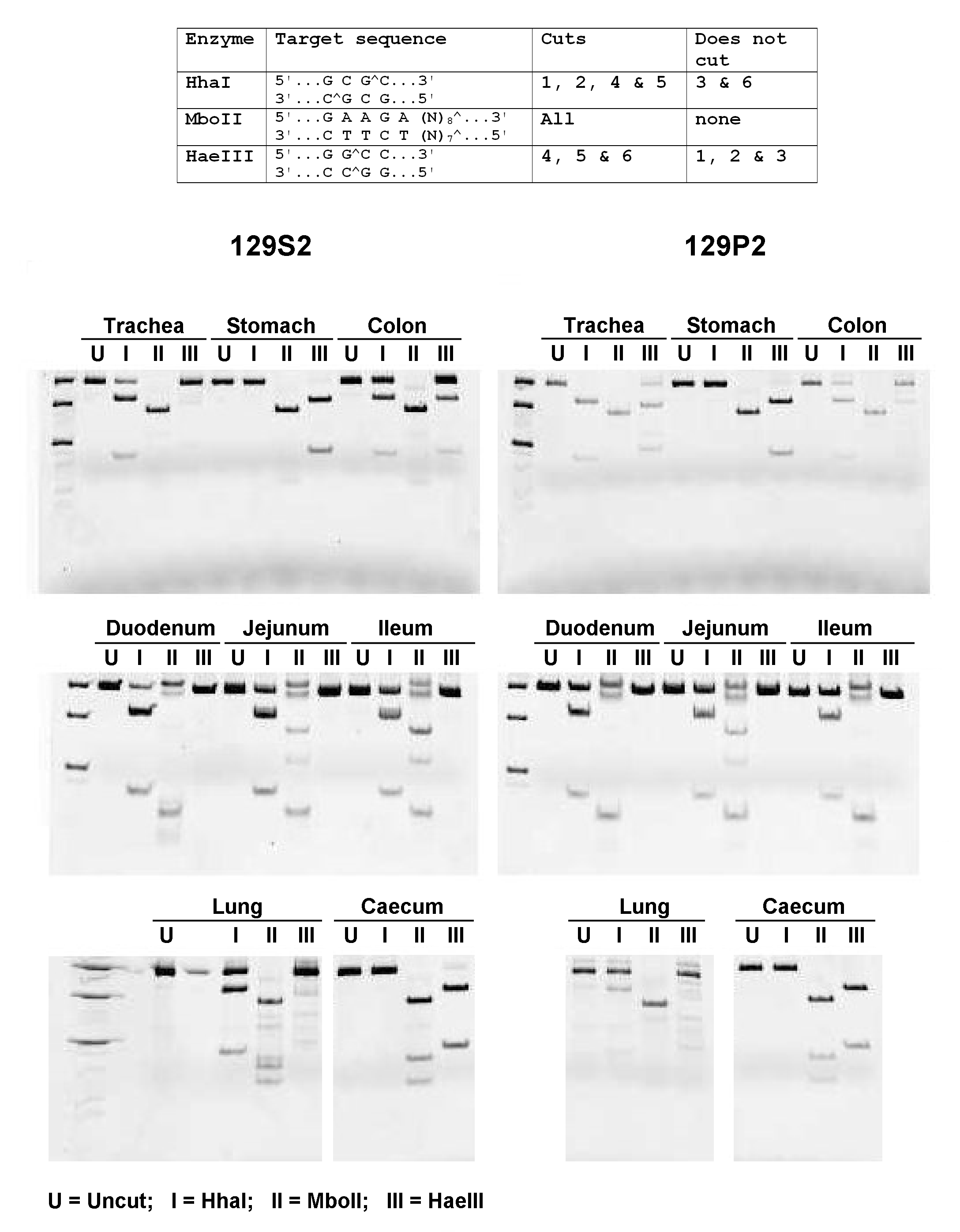

Supplement: Additional file 5 — Restriction enzyme analysis of Itln transcripts amplified from various tissues. Tissue specific expression of Itln variants in 129S2 and 129P2 mouse. [file 1471-2164-12-110-S5.PNG]
