# Supplementary material for: Strain-specific copy number variation in the intelectin locus on the 129 mouse chromosome 1
Source: BMC Genomics. 2011 Feb 16;12:110. doi: 10.1186/1471-2164-12-110 (PMC3048546; doi:10.1186/1471-2164-12-110)
Supplement: Additional file 2 — Genes in the Itln containing contigs of 129S7, C57BL/6J & Celera mouse. A list of accession numbers for the coding sequences on the three assemblies. [file 1471-2164-12-110-S2.PDF]

Table S1 – Genes in the *Itln* containing contigs of 129S7, C57BL/6J & Celera mouse.

Sequence	Accession Number (region)
Itln containing contig, 129S7/SvEvBrd-Hprt$^{b\text{-}m2}$	HM370554
Refbp2	ADJ67482
Itln6	ADJ67483
Cd244_SE	ADJ67485
Itln5	ADJ67484
Itln4 (frame-shift)	ADJ67486
Itln2 (Itlnb)	ADJ67487
Itln3 (truncated)	ADJ67488
Itln1 (Itlna)	ADJ67489
CD244	ADJ67490
Ly9	ADJ67491
Slamf7	ADJ67492
Itln containing contig, Ref. C57BL/6J Assembly	NC_000067 (Chr1:173431109-173531110)
Refbp2	NP_062357, ENSMUSP00000080242
Itln1 (Itlna)	NP_034714, ENSMUSP00000043837
CD244	NP_061199, ENSMUSP00000004829
Ly9	NP_032560, ENSMUSP00000069319
Slamf7	NP_653122, ENSMUSP00000056934
Itln containing contig, Celera Assembly	NW_001030662 (Chr1:85364426-85951426)
Refbp2	NP_062357
LOC640577 (Itln-like)	XP_922713
LOC677253 (Itln-like, frame-shift, truncated)	
LOC640587 (Itln-like, frame-shift)	
Itlnb	NP_001007553
LOC640605 (Itln-like, truncated)	
Itln1	NP_034714
LOC677008 (CD244)	XP_001003781
Ly9	NP_032560
Slamf7	NP_653122

GenBank's accession numbers for the contigs are that of the genomic sequences. Genes with coding sequences are represented by their GenBank accession numbers and, when available, ENSEMBL's (starting with EN) protein accession numbers.
