# Supplementary material for: Strain-specific copy number variation in the intelectin locus on the 129 mouse chromosome 1
Source: BMC Genomics. 2011 Feb 16;12:110. doi: 10.1186/1471-2164-12-110 (PMC3048546; doi:10.1186/1471-2164-12-110)
Supplement: Additional file 3 — 5' and 3' probes used in the Southern blot. Sequences of the Southern probes. [file 1471-2164-12-110-S3.PDF]

```
>5’probe

TCCTATGAATGATGATTATGGTAGATGTTAGGATTTTTTTAAGAGAAATATATAATT
TACAGGAAAGATACAAGCTGAAAACTAACACCCAACATCACCTCTTAGAATATGGAA
CAATATGGGTAGACCACACTGAATATTTGTCTTTTAGGGCCCACTAATCTCAGAATT
ACCCTGTAATAAGATGCTGTATCTACATAGGGCATCTTGCCAAGAGGAAAGTTTGTG
CCTCACTGGGCTTTCCCCATAAAAGGACCAAAGTCTCACACATGCAGGAGCCTCAGC
AGAGAAAGGTTCCTGCCATTACTCAGCTAGCAACTCTCAGCTCC

>3’probe
CAATGCTACCAGTGGAGAGGCTGCTGAGGTACAAAGAGTTCACTGAATTTGGCTATT
ATAGTGTCTTTAGACTCATTAGCTATAGCAGTCAGTCTTTTGCCTCTGTGATATATA
CTCAAGAGAAACAGGTTAAAGAAGAAAGTCTTATTTAGTTTCATAGATGTTAGCCTA
GGGTGAATTGGTTCCTCTGTTGTTAGATCAAGGGGGAGACAGAATCATCATACAGAA
AGGGCATATTAGAAGTGTGGCTTACCGAATGGTGACCAGGAAAGTGAGAGCCAGCAT
AAAAGGCCAGAGAAAATCATCTCCAAAAACATGCTCCTCTGTGGCTGAGGAGATAGA
TGAC
```

Figure S2 – 5’ and 3’ probes used in the Southern blot.
