# Supplementary material for: Strain-specific copy number variation in the intelectin locus on the 129 mouse chromosome 1
Source: BMC Genomics. 2011 Feb 16;12:110. doi: 10.1186/1471-2164-12-110 (PMC3048546; doi:10.1186/1471-2164-12-110)
Supplement: Additional file 4 — Detection of Itln CNV in non-C57/BL mouse strains using next-generation sequencing. Plots of log2 ratio and sequencing coverage at the Itln locus of different mouse strains. [file 1471-2164-12-110-S4.PDF]

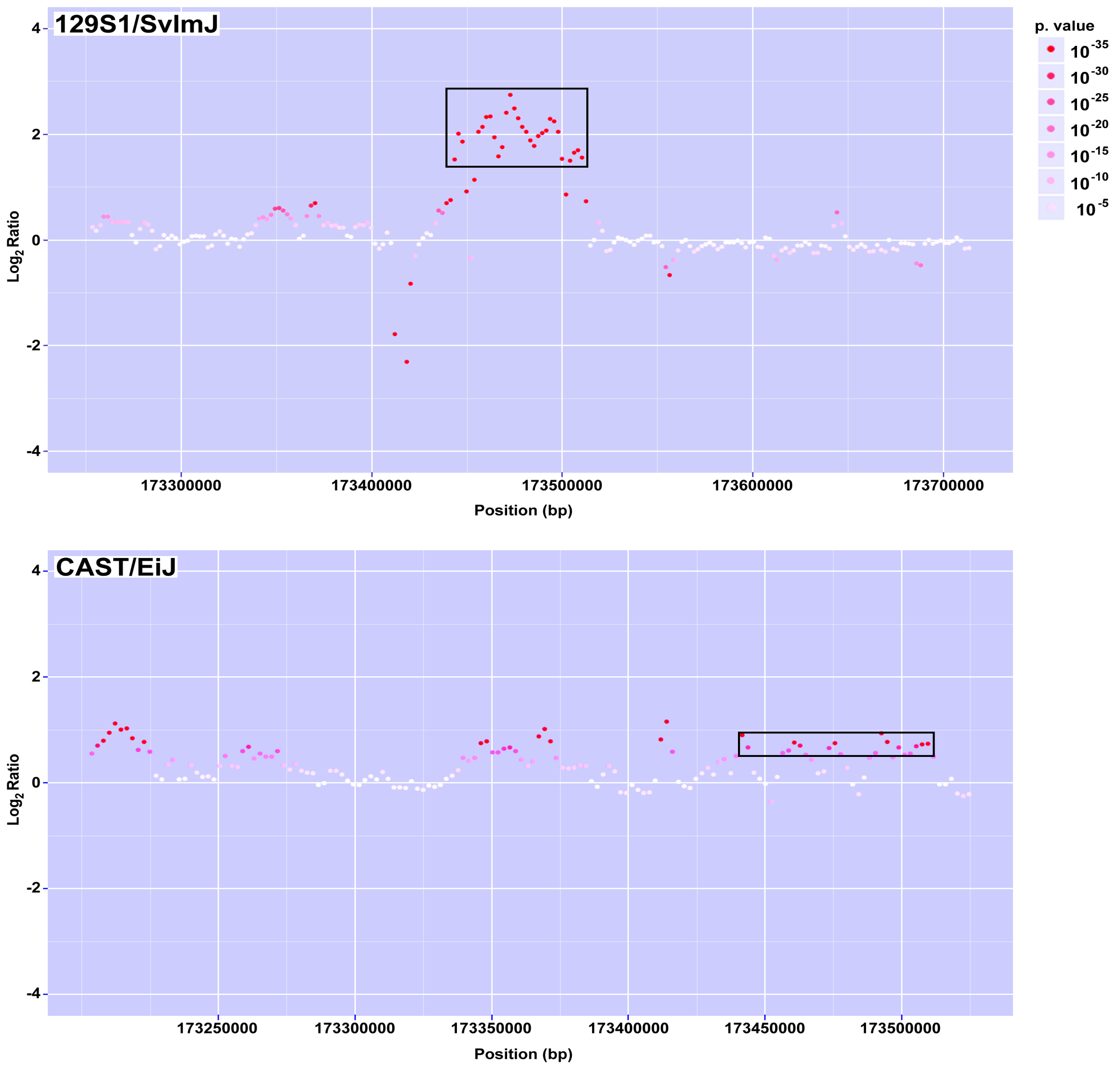
129S1/SvImJ
CAST/EiJ
p. value
10^{-35}
10^{-30}
10^{-25}
10^{-20}
10^{-15}
10^{-10}
10^{-5}
Log_2 Ratio
Position (bp)
173300000
173400000
173500000
173600000
173700000
173250000
173350000
173450000
4
2
0
-2
-4

Figure S3a

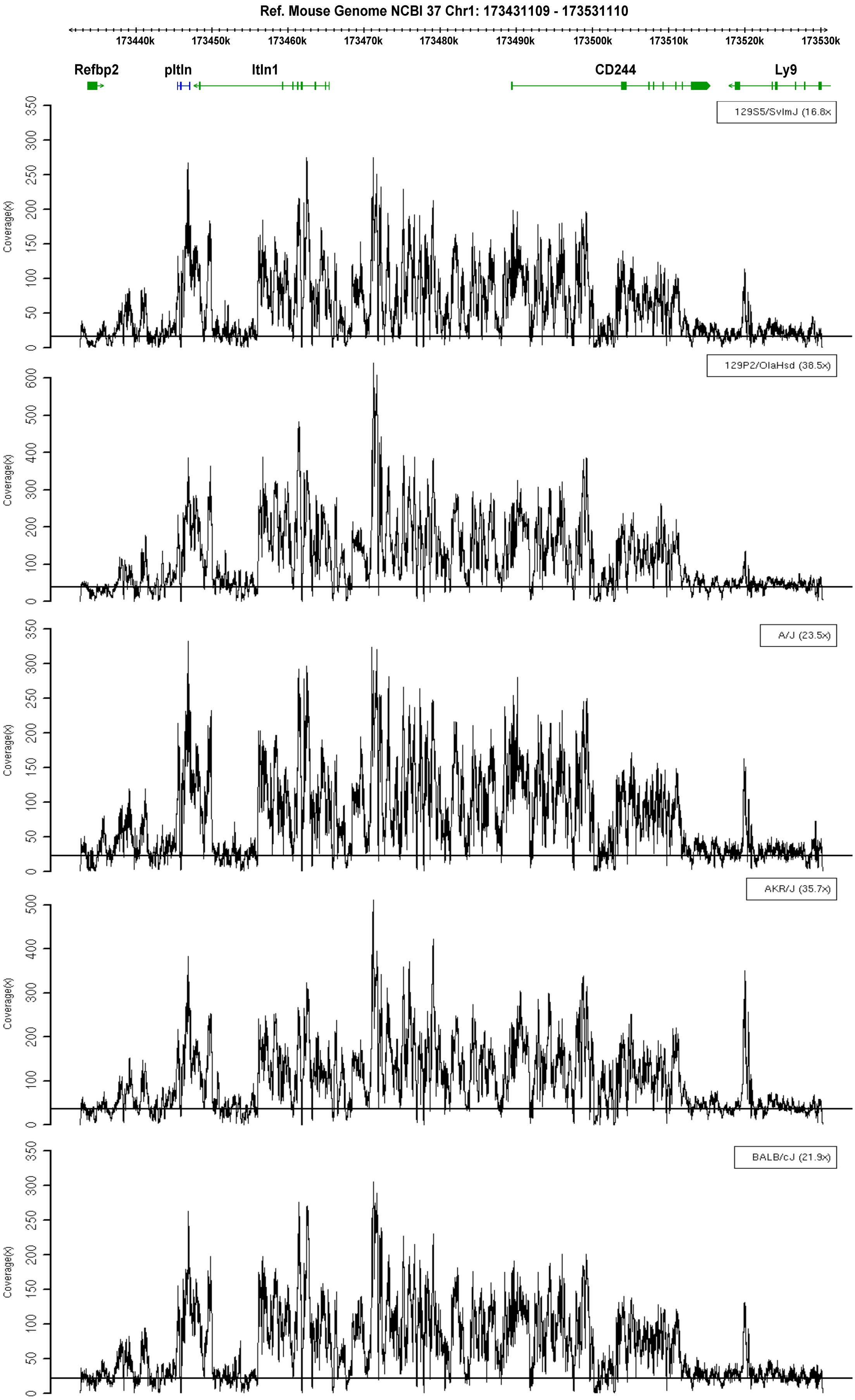

Figure S3b (cont…)

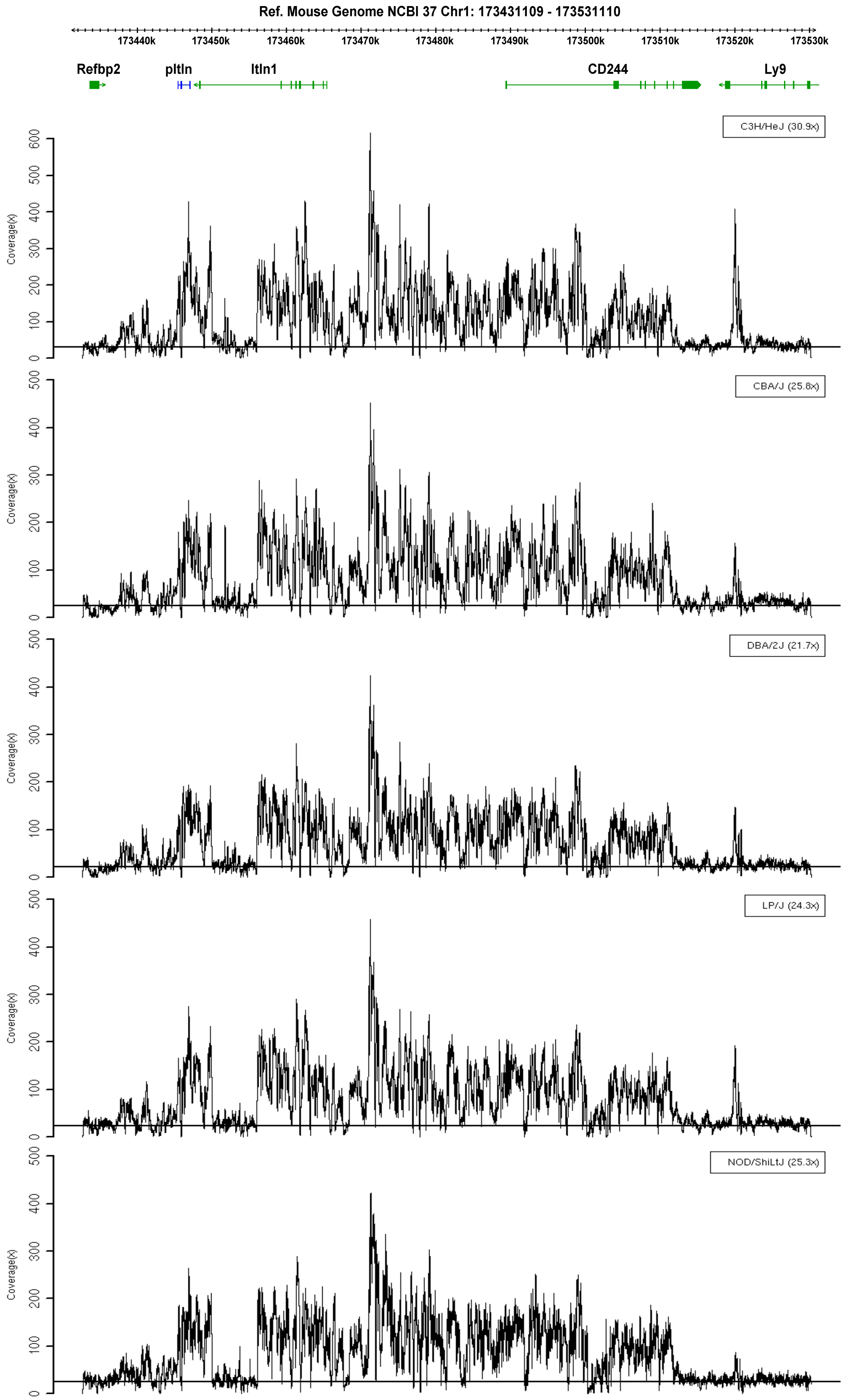

Figure S3b (cont…)

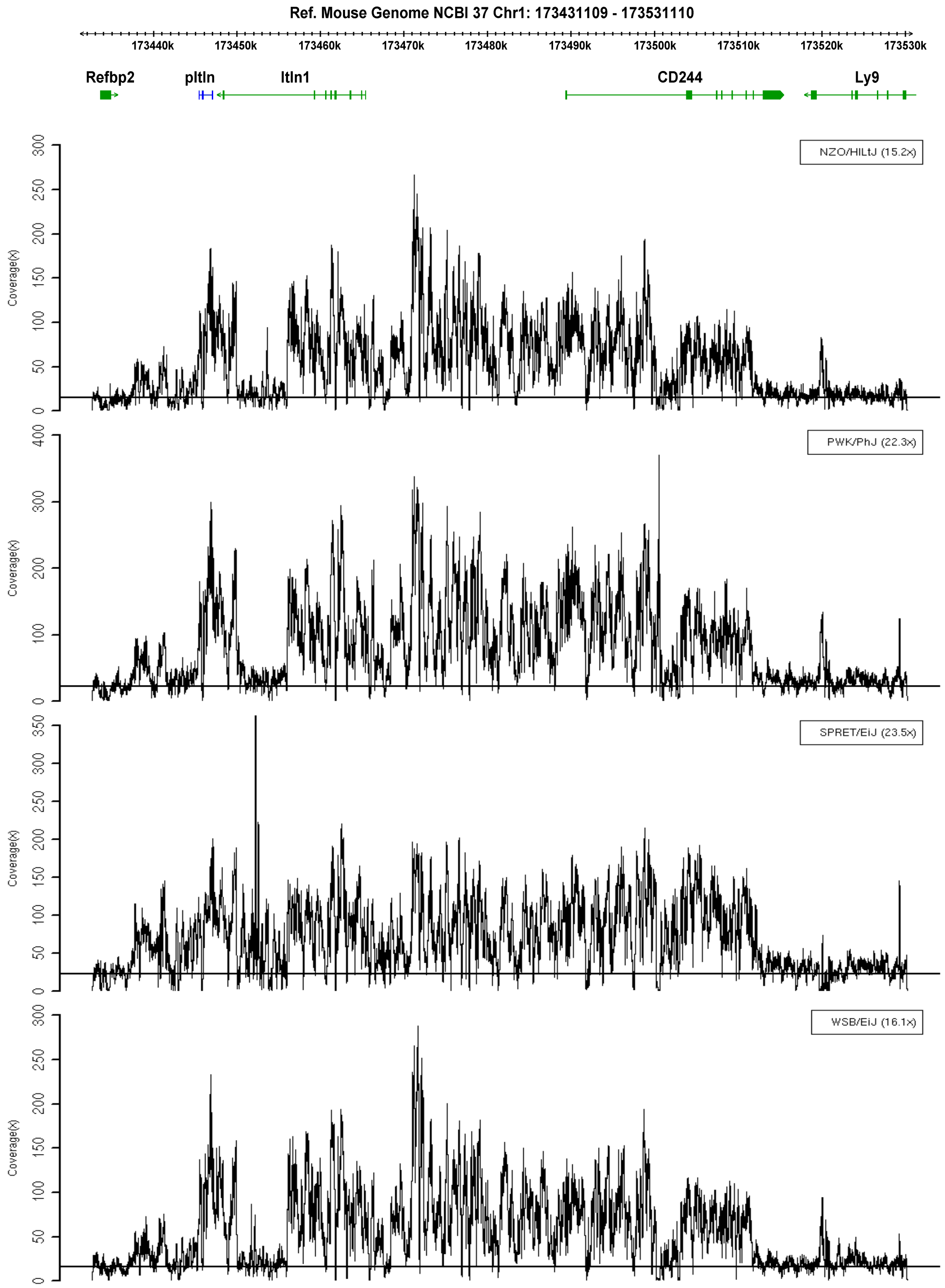
Ref. Mouse Genome NCBI 37 Chr1: 173431109 - 173531110
173440k
173450k
173460k
173470k
173480k
173490k
173500k
173510k
173520k
173530k
Refbp2
pltln
Itln1
CD244
Ly9
NZO/HILtJ (15.2x)
PWK/PhJ (22.3x)
SPRET/EiJ (23.5x)
WSB/EiJ (16.1x)
Coverage(x)

Figure S3b

Figure S3 – Detection of *Itln* CNV in non-C57/BL mouse strains using next-generation sequencing.

(a) Log_2 ratio plot of CNVs on the Chr1 of the different mouse strains. The duplicated *Itln* locus on the plot is boxed. 2 plots were presented here as the other plots resemble that of the 129S1. (b) Coverages of the paired-end reads along the *Itln* locus were plotted. Horizontal line across each plot indicates the mean mapped coverage of the genome. Regions of sequence duplication/expansion resulted in the higher than average coverages.
